# Supplementary material for: ChatGPT as a Tool for Biostatisticians: A Tutorial on Applications, Opportunities, and Limitations
Source: Stat Med. 2025 Oct 23;44(23-24):e70263. doi: 10.1002/sim.70263 (PMC12548020; doi:10.1002/sim.70263)
Supplement: Supplementary file 1 — Data S1. Supporting Information. [file SIM-44-0-s001.zip › Supplementary Materials Additional.zip!/3.1_Systematic_Reviews_and_Meta_aspects/3.1_Data Extraction - o4-mini - Chat 2.pdf]

Use Case Meta-Analysis - Data Extraction - o4-mini - Chat 2

Rudloff et al. - 2009 - Concurrent lobular neoplasia increases the risk of ipsilateral breast cancer recurrence in situ treated with breast-conserving therapy.pdf
PDF

Vidali et al. - 2012 - Conservative treatment of breast ductal carcinoma in situ results of an Italian multi-institutional.pdf
PDF

The aim is to perform a meta-analysis on total ipsilateral local recurrence rates at 10 years in cases of ductal carcinoma in situ.

Data inclusion rules are: To ensure the validity of the follow-up data, the results should be as close to 10 years follow-up (minimum) as the information in each study allows. If there is more than one publication from an institution or group, the most recent study with appropriate follow-up will be used to extract 10-year data.

Please provide from each of the two attached studies:

- the rate of ipsilateral local recurrence for conservative surgery with additional radiotherapy (no other interventions or drugs allowed) after 10 years

- the number of eligible patients

- period of data collection

- duration of follow-up

- type of study

- age of patients (mean or median, maximum and minimum)

- treatment modalities

For this task you have access to up-to-the-minute web retrieval to find adequate approaches for data extraction. When answering the task above please proceed as follows:

1. Fetch the most relevant sources.

2. Show your step-by-step reasoning.

3. At the end, give me a concise answer citing the sources you used.

4. Please provide a dataframe with one entry for each study, and column names "study", "rate", "n_patients", "period", "follow_up", "study_type", "age", and "treatment".

Step-by-step reasoning

1. **Identify the two studies**
    - **Rudloff et al. (2009)**: “Concurrent lobular neoplasia increases the risk of ipsilateral breast cancer recurrence in patients with ductal carcinoma in situ treated with breast-conserving therapy”
    - **Vidali et al. (2012)**: “Conservative treatment of breast ductal carcinoma in situ: results of an Italian multi-institutional retrospective study”
2. **Extract for Rudloff et al.**
    - **Eligible patients**: 294
    - **Period of data collection**: 1991–1995
    - **Duration of follow-up**: Median 11 years (range 0–16 years)
    - **Type of study**: Retrospective cohort review of institutional database
    - **Age of patients**: Median 55 years (range 26–89 years)
    - **Treatment modalities**: Breast-conserving surgery (excision) plus adjuvant external-beam radiotherapy; no cytotoxic chemotherapy; tamoxifen in 6.1%
    - **10-year ipsilateral recurrence rate (surgery + RT)**: 11.9%
3. **Extract for Vidali et al.**
    - **Eligible patients**: 586
    - **Period of data collection**: February 1985–March 2000
    - **Duration of follow-up**: Median 136 months (≈11.3 years; range 16–292 months)
    - **Type of study**: Multi-institutional retrospective cohort study
    - **Age of patients**: Median 55 years (range 29–84 years)
    - **Treatment modalities**: Conservative surgery (quadrantectomy/tumorectomy/wide excision) + whole-breast RT (median 50 Gy; boost in 50% cases to median 10 Gy)
    - **10-year ipsilateral recurrence rate**: 9.6%

Concise answer

study	rate	n_patients	period	follow_up	study_type	age	[illegible]
Rudloff et al. (2009)	11.9 %	294	1991–1995	Median 11 y (0–16)	Retrospective cohort review	Median 55 y (26–89)	[illegible]
Vidali et al. (2012)	9.6 %	586	Feb 1985–Mar 2000	Median 136 mo	Multi-institutional retrospective	Median 55 y (29–84)	[illegible]

study	rate	n_patients	period	follow_up	study_type	age	
							b<br>1

Quellen
